# Exploring the relationship between EFL students’ writing performance and activity theory related influencing factors in the blended learning context

**DOI:** 10.1371/journal.pone.0305668

**Published:** 2024-06-17

**Authors:** Yuling Wang, Shaidatul Akma Binti Adi Kasuma, Salasiah Binti Che Lah, Qi Zhang

**Affiliations:** 1 School of Languages, Literacies and Translation, Universiti Sains Malaysia, Penang, Malaysia; 2 School of Foreign Languages and Literatures, Chongqing University of Education, Chongqing, China; Bahir Dar University, ETHIOPIA

## Abstract

With the rapid development of technologies, blended learning is widespread in English writing instruction. The effect of blended learning on EFL writing outcomes is affected by various factors. This study examines the relationship between EFL students’ writing performance and influencing factors and the relationship between these factors in a blended learning context based on the Activity Theory. The study used a quantitative method: English argumentative writing tests and questionnaires with 33 undergraduates. The results reveal that EFL students’ argumentative writing performance is significantly and positively correlated with five influencing factors, in descending order of correlation: subject, community, object, rules, and division of labor. Moreover, the findings suggest a significant positive relationship within each factor in the blended learning environment, except for no relationship between rules and division of labor. Furthermore, the research provides useful references and insights for further research and educational practice in blended writing instruction. Due to limitations such as the relatively small sample size, the focus on argumentative writing, and the reliance on quantitative data, this study gives the impression that the results only represent a portion of the population and situation. Therefore, future research could consider enlarging the sample size, adopting a more comprehensive range of writing genres, involving qualitative methods, or expanding the scope of research on the impact of BL on other disciplines.

## 1. Introduction

Writing is the most challenging of the four basic linguistic skills (listening, reading, speaking, and writing) [1]. The capacity to master writing skills reflects students’ comprehensive ability of language use, so the instruction of writing is an essential component of English as a Foreign Language (EFL) teaching. However, traditional EFL writing instruction is one-time writing, meaning the initial draft is the final [2]. Teachers are the only readers of students’ writing texts, making students accustomed to receiving teachers’ guidance and feeling that they are writing for teachers [3]. In the meantime, the number of writing texts that need to be corrected is too large for teachers, which leads to untimely feedback [4,5]. As a result, teachers spend much time correcting compositions, but the effect of EFL writing teaching is still not ideal [4].

Blended learning (BL) aims to maximize teaching results by combining various resources and methods [6,7]. Through online learning platforms, BL encourages students to carry out synchronous and asynchronous interactions and provides a potentially feedback-rich environment, enhancing student learning outcomes and fostering a continuous feedback culture [8–13]. To address the dilemma of traditional teaching, online learning platforms were introduced into the field of tertiary education [14–16], and BL has increased at a rapid rate since the early 21st century [17].

Despite students holding a positive attitude towards the BL in language learning [18–23], the effectiveness of blending face-to-face instruction and online learning is affected by both the merits and demerits of the two learning modes [17]. Previous studies [24–28] indicate that students are impacted by several factors in the BL settings, such as motivation, self-regulation skills, and network environment. Moreover, some research focused on time management and teamwork skills in BL mode [29,30].

Unlike the previous research, we designed survey items for five factors based on the Activity Theory (AT) [31]. The five factors, including the subject (the person who writes), the object (the purpose or task of writing), the community (the social environment in which the writing takes place), the rules (the norms and standards), and the division of labor (the distribution of tasks and responsibilities in writing activities), contribute to an understanding of EFL students’ writing activities. These factors interact with each other to form a dynamic system that affects the writing process and outcome [31]. Through analyzing these factors, we can gain insight into the nature of the writing activity and what influences the writing outcome in BL contexts.

Though few studies have attempted to investigate the relationship between EFL writing performance and various influencing factors, as far as researchers’ knowledge is concerned, no research has been conducted on the relationship between EFL students’ writing performance and the influencing factors in BL contexts based on Activity Theory. Thus, this study examines the relationship between five influencing factors (subject, object, rules, community, and division of labor) and EFL students’ writing performance, and it also explores the relationships among these influencing factors in a blended learning context. This research may help educators and teachers further understand the influencing factors of BL and provide information for instructors to design more efficient lessons of EFL writing. Also, the research results can provide a scientific basis for policymakers to promote the optimization of BL in EFL writing instruction.

## 2. Literature review

### 2.1. Blended EFL writing learning

The original BL refers to the study mode integrating online learning and face-to-face teaching [32]. To meet trainees’ desires regarding place and time, enterprises explored the mix of online and face-to-face training [33]. Subsequently, this teaching mode was implemented step by step in higher education, as well as in EFL writing instruction. Previous studies [6,27,34] have demonstrated the overall effectiveness of BL on EFL writing.

Artifacts like the internet, videos, computers, mobile, and online resources are usually used by teachers to improve EFL students’ writing outcomes. According to Afilina’s [35], Karo et al.’s [36], and Sianna’s [37] findings, employing these artifacts can provide a comfortable and active writing atmosphere, increase self-confidence, and improve learning outcomes. Similarly, Park and Jung’s [38] study indicates that using videos in EFL writing instructions can stimulate students’ motivation, participation, and overall achievement. Also, Andres et al. [39] concluded that using videos with cultural content in EFL writing courses can improve vocabulary, transitional words, punctuation, linguistic structures, and ideas. Moreover, Rahimi and Fathi [40] engaged Wiki in an EFL writing course and found that it can improve writing performance, self-regulation, and self-efficacy. Therefore, involving online resources in traditional face-to-face teaching is suitable for EFL writing instruction.

Although studies about blended EFL writing learning centred around the advantages of BL, there are some challenges. For instance, compared with face-to-face instruction, BL puts forward higher requirements for students’ ability to self-regulate and learn autonomy [41]. Especially in online environments, students must arrange their learning progress reasonably and self-regulate to avoid distractions from information technology and network resources [28]. In addition, students sometimes face difficulties when conducting blended learning because of unequal access to technology and networks in different regions [42]. Some students cannot connect to the internet at home due to device issues and the lack of technology skills, which make online materials inaccessible [43]. According to Xavier and Menses’ [44] study, BL suffers from the disadvantages of students’ overloading and high dropout rates in tertiary BL courses. Furthermore, Szadziewska and Kujawski [45] reported that students perceived drawbacks of BL, such as log-in and download problems, lower motivation to learn, insufficient materials available, no solutions to tests and tasks, user-unfriendly interface, lower creativity in searching for knowledge, and no direct communication with other participants.

Moreover, as sociocultural interactions are vital to learning [46], technologies are often employed in language teaching to create a BL environment and promote EFL writing skills [40,47–51]. Jiang and Zhang’s [52] study indicates that mobile-assisted collaborative writing environments can provide explicit interactions and facilitate EFL argumentative writing performance. However, Lin et al. [53] investigated the effect of Augmented Reality (AR) applications in a writing course. They concluded that employing technologies is beneficial to long-term memory, motivation, and self-regulation in EFL writing, but it led to mixed results in writing achievements. The effect of BL on EFL writing performance may be good or bad and may be affected by many factors.

Although the previous studies [23,54–57] indicate that the use of BL is effective in improving writing performance by giving students more chances to participate, increasing the obligation to learn, exposing them to online materials, and providing interactions with others outside the classroom, few studies have analyzed the extent to which influencing factors in BL environment affect EFL writing performance. Therefore, exploring the relationship between the influencing factors and EFL writing performance in BL contexts is necessary to achieve better BL outcomes and provide valuable suggestions for the pedagogical practice in EFL writing courses.

### 2.2. AT

AT is a philosophical and interdisciplinary framework that describes the meaningful behaviors of a person or a group and relates the behavior to the context. From the perspective of AT, learning is regarded as a symbolic procedure or intermediary effect in which students proactively build their knowledge environment and engage in target-oriented activities. AT evolved from Vygotsky’s Sociocultural Theory, and the development of AT contains three generations. Vygotsky, Leont’ev, and Engeström are the representatives of the three stages and have made a breakthrough in the development of AT.

According to Vygotsky [46], human inner psychological activities cannot be separated from external behaviors and related social environments, which means the psychological and social structures interact. The construction process of personal knowledge is inseparable from the knowledge sharing of the social group.

Based on Vygotsky’s theory, Leont’ev clarified the boundary between individual and collective behavior [58]. One of Leont’ev ’s most vital contributions to the AT is that he proposed the unit of analysis in Activity Theory. He believes systematic analysis of human behavior should be divided into hierarchical levels: activity, action, and operation [59].

Engeström [31] argued that neither Leont’ev nor Vygotsky’s AT ultimately revealed the essence of the activity system. He expanded the activity model by adding rules, community, and division of labor. The bottom of Engeström’s Activity Model is composed of rules, community, and division of labor, which forms the conceptual framework integrating individual activities with society, culture, and history. Hence, activities are no longer regarded as single interactions between subject and object but collective activities.

**Fig 1:**
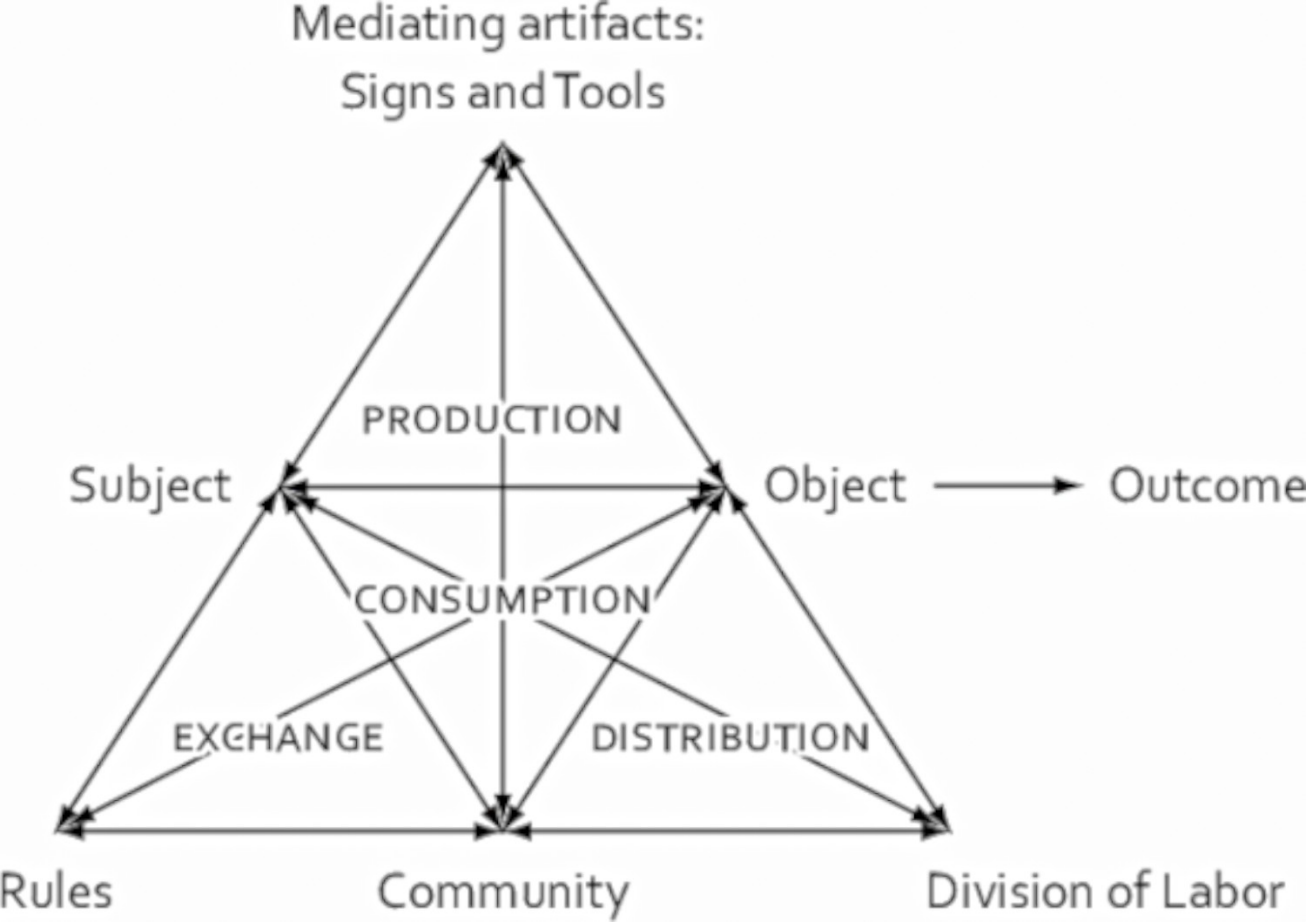
Engeström’s Activity Model [31]. This figure illustrates Engeström’s Activity Model, which includes six elements: mediating artifacts (signs and tools), subject, object, rules, community, and division of labor. Mediating artifacts is at the top. The subject and object are in the middle. Rules, community, and division of labor are at the bottom. These elements are connected by arrows representing dynamic relationships to form a triangle. They interact with each other and ultimately influence the outcome.

According to Engeström’s Activity Model, an activity system consists of six interactive elements (subject, object, mediating artifacts/tools, rule, community, and division of labor), which constitute four sub-systems (production, consumption, exchange, and distribution), as shown in Fig 1. This model places human activities in a specific sociocultural environment and provides an operable analytical framework with six elements.

The subject, often the core of research, can be individuals or groups. The object refers to the original materials or problem to which this activity is directed, which would finally be transformed into a specific outcome. Activities are influenced and restricted by rules, community, and division of labor. The community includes the participants with the same goal as the subject, and the object simultaneously impacts the subject and the ongoing collective activity. The rules mean explicit or implicit habits, standards, and social relations constraining the subject’s actions. The division of labor means the horizontal action and interaction between community members and the vertical division of authority and position. Engeström’s Activity Model [31] shows the factors and components of activities and the connections of these elements.

Blended EFL writing learning could be analyzed from an AT perspective, as it can be conceptualized as a collective and contextual activity [60]. AT has been used in EFL writing research, such as studies on the influence of feedback in improving EFL students’ writing performance [61,62], students’ writing strategies [63], and factors affecting EFL writing [64,65]. As for research in BL contexts, Pullenayegem et al. [66] adopted AT to investigate the influencing factors that English students met in their writing courses, indicating that the strict rules and the number of rules required to be followed interfered with interactions and cooperation between students. Moreover, Hajimaghsoodi and Maftoon [67] used an e-learning platform to design a language learning framework based on AT for English writing courses and surveyed its effect. The results showed the positive impact of the AT-based language learning framework in second language (L2) writing classes, and they suggested students interact with tools, communities, division of labor, and rules to achieve better learning performance.

In this study, AT is used as a lens through which to explain and demonstrate the findings. Since the current research focuses on the factors influencing EFL writing performance in a BL environment, rather than focusing on the impact of specific instruments/technologies that support the BL, the factor of tools was not included as one of the factors examined in this research. This study included five influencing factors: subject, rules, community, division of labor, and object.

- Subject: EFL students who are the actors of blended writing activities.
- Object: Finishing argumentative writing texts and improving writing performance, which are the goals or tasks of the blended writing activities.
- Rules: The constraints in blended writing activities, such as time constraints, anonymous policy, automatic evaluation criteria, grading standards, and group rules.
- Community: The social environment in which the activities take place and are influenced, such as teachers and classmates involved in the activities.
- Division of labor: The teachers’ and students’ specific responsibilities and relations in blended writing activities, such as teacher and peer feedback.

The five factors mentioned above influence EFL students’ writing outcomes in the blended writing activities. The EFL students are influenced by the writing rules and regulations when completing argumentative writing tasks in BL contexts. Teachers and classmates, who are community members, provide EFL students with rich interactions and authentic learning contexts that impact their writing development. In addition, the division of labor, such as teacher feedback and peer feedback, directly affects the quality of students’ writing by providing suggestions and collaborative learning, which in turn influence writing outcomes. These factors interact with each other to influence EFL writing performance in BL environments.

## 3. Methodology

The present study used a quantitative method to examine the relationship between EFL writing performance and five influencing factors in a BL context of face-to-face instruction and online activities based on the WeChat application and Pigai.org online writing platform.

### 3.1. Research design

The main study utilized a correlational research design and adopted AT to explore the correlation between influencing factors and EFL students’ writing performance in BL settings in terms of subject, rules, community, division of labor, and object. This research has one dependent variable and five independent variables. The samples were selected by utilizing a cluster sampling technique. They were from one class in the field of English at a university (Yangtze Normal University) in China. Although the samples are limited to one class, every effort was made to consider inclusivity in global research in the study design. This study received a waiver for ethical approval from the Human Research Ethics Committee of Yangtze Normal University (Ref No. R2023-344665). Additional information regarding the ethical, cultural, and scientific considerations specific to inclusivity in global research is included in the Supporting Information (S1 Checklist). The chosen EFL students’ (n = 33) writing performance, which was collected from the argumentative writing test, constituted the dependent variable. At the same time, the five influencing factors assessed through a five-point Likert scale questionnaire were the independent variables. The study was conducted in the following four phases: a) carried out a pilot study to evaluate the reliability and validity of the instruments and made appropriate modifications to them; b) implemented ten weeks of blended English writing instruction, which integrated classroom teaching and online learning among EFL undergraduate students. This duration enabled researchers to observe not only short-term outcomes but also potential longer-term effects or sustained changes resulting from the intervention [65,68]; c) argumentative writing tests and questionnaires were administered to collect quantitative data; d) analyzed the data with Statistical Package for the Social Sciences (SPSS) and reported research findings.

### 3.2. Population and sample size

The population of the present study is Chinese EFL undergraduate students. We took a week (between 1^st^ and 7^th^ March 2023) to recruit participants from a general undergraduate university located in the southwest of mainland China, Yangtze Normal University, which recruits students from 91% of China’s provinces and cities so that the subjects are representative. The participants provided signed informed consent forms before starting the study.

As this study used Pearson Correlation to analyze the quantitative data, the sample size should meet the required minimum sample size. According to the sample size calculation for the Pearson Correlation [69], the following formula gives the minimal sample size of seventeen participants when choosing the most frequent significance level (*α* = 0.05), the ideal statistical power (1−β = 0.8), and the large effect size (δ = 0.8) and assuming that the overall variance (σ2 = 1) and correlation coefficients (ρ = 0.5) between variables are chosen as conservative estimates [70–72].


$$n_{r}=\left(\frac{{\left({Ζ}_{1-\alpha /2}+{Ζ}_{1-\beta }\right)}^{2}}{\delta^{2}\times (1-\rho^{2})}\right)$$

Using a cluster sampling technique, this study selected a natural class of 33 students as participants in the main research. They were undergraduates majoring in English and enrolled in the English writing course, a mandatory 2-credit course designed for first-year undergraduate EFL students. Among them, 28 are females and 5 are males.

Moreover, 30 undergraduates (21 females and 9 males) majoring in English from different grades and classes at the same university, who did not take part in the main study, participated in the pilot study. Although the participants were at the same research site, they were on campus for different semesters and did not have the opportunity to meet one another during this study.

### 3.3. Data-gathering instruments, data collection and analysis

The current study used two data-gathering instruments to collect the data: the English argumentative writing test and the questionnaire. After a ten-week blended English writing course (from 13^th^ March 2023 to 26^th^ May 2023), a quantitative method was utilized for data collection on 2^nd^ June 2023, including questionnaires (S1 Appendix) and writing tests (S2 Appendix). The English argumentative writing test required participants to compose an argumentative essay on one of the provided topics within 45 minutes, the same as the writing time in the Test for English Majors-level 4 (TEM-4). The writing test provided four topics related to students’ study and life to ensure that students could choose familiar topics for argumentative writing, such as who should arrange children’s spare time activities, students should spend more time in clubs/sports or studies, different friends or similar friends, and whether it is better to live in their hometown or another city after graduating from university. The assessment criteria for writing performance (S3 Appendix) were adapted from Brown and Bailey [73], Hajimaghsoodi and Maftoon [67], and Jacobs [74], based on a 100-point scale, encompasses five aspects: content (30 points), style (20 points), language use (25 points), organization (20 points), and mechanics (5 points).

According to Coombe [75], the recommended number of evaluators is two, with a third in case of extreme disagreement. Two instructors with more than five years of English writing instruction experience were invited to rate the students’ writing tests, and a PhD candidate in applied linguistics as an alternate evaluator in case of disagreement. The researcher briefed the evaluators on the research design before they commenced scoring. Subsequently, the researchers used a day to train the evaluators. During the training, we comprehensively explained the scoring criteria and the scoring form on which they fill in the scores. We also established benchmarks using two argumentative compositions written by EFL undergraduate students. Then, the evaluators were asked to score two argumentative compositions, and we calibrated scoring to ensure the consistent evaluation of argumentative writing performance.

The questionnaire was designed based on AT [31] and adapted from Dwihandini et al. [76], Hajimaghsoodi and Maftoon [67], Portnov-Neeman and Barak [77], and Zeng [78]. The questionnaire items were modified to simplify and clarify the language and item structure to make the questionnaire more meaningful and contextually relevant to the respondents. For instance, all the items have changed from past to present. The clarification of wording has been improved, such as “blended learning” and “online platform” being changed to “this English writing course” and “Pigai.org”, respectively. As the participants were English majors, the questionnaire items were all presented in English, which was acceptable for participants. To guarantee respondents’ engagement and reduce response fatigue, the questionnaire was constrained to a maximum of 25 questions [79,80]. Table 1 presents details of the questionnaire.

**Table 1:** Details of the questionnaire.

Section	Variables	Number of Items	Source
**I**	Demographic information	-	-
**II**	Subject	5	Dwihandini et al. [76]
	Rules	5	Four items from Hajimaghsoodi and Maftoon [67] One item from Portnov-Neeman and Barak [77]
	Community	5	Hajimaghsoodi and Maftoon [67]
	Division of labor	5	Two items from Zeng [78] Two items from Portnov-Neeman and Barak [77] One item from Hajimaghsoodi and Maftoon [67]
	Object	5	Three items from Portnov-Neeman and Barak [77]

The first section of this questionnaire is demographic information. The second section of this questionnaire employed a 5-point Likert scale ranging from 1 (Strongly disagree) to 5 (Strongly agree) and closed-ended questions. The key merit of closed-ended questions is their direct nature, leaving no subjective space for evaluators [81]. Uniformly assigning five questions to each theme ensured equitable data collection on the five variables, mitigating the potential for bias favoring any factor.

The main study has collected writing scores and questionnaires after a ten-week blended EFL writing course. All collected data were inputted into SPSS version 26 for data cleaning and analysis. Scores and responses from 33 students were analyzed, and there were no missing data. In addition to using Pearson Correlation to analyze the data, descriptive statistics such as mean, standard deviation, and frequency were employed in this study.

### 3.4. Pilot study

The pilot study in this research aimed to evaluate the reliability and validity of the instruments (writing tests and questionnaires) and make any necessary modifications to improve the instruments.

#### 3.4.1 Pilot-testing of the writing test

Cohen’s Kappa analysis was used in the pilot study to assess the inter-evaluator reliability of the writing test. According to Landis and Koch [82], the results (Cohen’s kappa = 0.72, P<0.001) indicate a substantial agreement between the two evaluators in scoring. Therefore, inter-rater reliability was achieved between the raters. Moreover, the reliability of the writing test in the pilot study was assessed using the Cronbach Alpha. The result showed that Cronbach’s Alpha Coefficient (0.93) was high, indicating that the writing test is reliable.

#### 3.4.2 Pilot-testing of the questionnaire

The reliability and validity of the questionnaire were also investigated. To determine whether the questionnaire had a proper level of internal consistency and Split-half reliability, this study used Cronbach’s α and Spearman-Brown prophecy formula in SPSS. The total scale of Cronbach’s α was 0.877, and the Split-half reliability coefficient was 0.734, indicating that the reliability of this questionnaire is at an accepted level [83,84].

The validity of the questionnaire is usually reflected by the indicators of content validity, construct validity, convergent validity, and discriminant validity. To ensure content validity, the questionnaire items were revised many times according to the advice given by three instructors who have more than ten years of experience in tertiary EFL writing teaching. The main revision suggestions proposed by the three experts were summed up as follows. Firstly, the tense of all items should be changed from past to present tense. Secondly, each item should be presented in a concise and non-wordy way. Finally, the “blended learning” or “blended writing course” should be changed to “this course” to allow respondents to understand the meanings of survey items clearly.

To verify the construct validity, convergent validity, and discriminant validity, this research employed Confirmatory Factor Analysis (CFA) in SPSS to test the items’ loadings, Cronbach’s alpha (α), composite reliability (CR), and average variance extracted (AVE) of each variable. As presented in Table 2, each variable’s loadings (between 0.501 and 0.961) have reached the criterion of 0.5, and all the CR (between 0.811 and 0.899) were above 0.7, suggesting adequate construct validity [85]. Although the variables’ AVE (ranging from 0.479 to 0.649) were not all greater than 0.5, convergent validity could still be accepted as the loadings were greater than 0.5 [86].

**Table 2:** Results for pearson correlation analysis of the five variables in the pilot study.

Variables	Items	Loadings	Cronbach’s α	CR	AVE
Subject	1	0.646	0.820	0.819	0.479
	2	0.605			
	3	0.659			
	4	0.769			
	5	0.774			
Rules	6	0.823	0.794	0.811	0.481
	7	0.511			
	8	0.707			
	9	0.749			
	10	0.534			
Community	11	0.648	0.891	0.899	0.649
	12	0.839			
	13	0.931			
	14	0.754			
	15	0.772			
Division of Labor	16	0.748	0.799	0.816	0.483
	17	0.880			
	18	0.689			
	19	0.501			
	20	0.579			
Object	21	0.569	0.831	0.837	0.513
	22	0.961			
	23	0.605			
	24	0.668			
	25	0.769			

Note: AVE = Average Variance Extracted. CR = Composite Reliability.

Discriminant validity was assessed by comparing the AVE square root with the correlations between each pair of variables. As shown in Table 3, each variable’s AVE square root is greater than its correlation coefficient with other variables, indicating that the constructs have sufficient discriminant validity [86].

**Table 3:** Results for AVE square root values and pearson correlation between variables.

	Subject	Rules	Community	Division of Labor	Object
Subject	**0.692**				
Rules	0.159	**0.694**			
Community	0.358	0.274	**0.806**		
Division of Labor	0.441	0.194	0.490	**0.695**	
Object	0.474	-0.041	0.258	0.384	**0.716**

Note: Bold numbers = square root of the AVE. The lower left values = correlations between variables.

## 4. Results

### 4.1 Descriptive statistical analysis of the items

Table 4 presents each questionnaire statement’s means, standard deviations, and frequencies. At the bottom of each factor, the average mean scores, standard deviations, and average frequencies are provided. The average value of the object (M = 4.39, SD = 0.36) was the highest among all the influencing factors, with rules (M = 3.97, SD = 0.40) and community (M = 3.97, SD = 0.36) tied for second place followed by subject (M = 3.86, SD = 0.39) in third place and division of labor (M = 3.80, SD = 0.30) at fourth.

**Table 4:** Means, standard deviations, and frequencies for each item.

Influencing Factors	Item No.	Mean (M)	Std. Deviation (SD)	Frequencies (F)		
				Disagree	Neither Agree nor Disagree	Agree
Subject	1	4.09	0.58	0	4	29
	2	3.64	0.65	1	12	20
	3	3.79	0.74	2	7	24
	4	4.06	0.70	1	4	28
	5	3.73	1.04	6	5	22
	Average	3.86	0.39	2	6.4	24.6
Rules	6	4.00	0.61	0	6	27
	7	4.03	0.77	0	9	24
	8	4.12	0.74	0	7	26
	9	3.91	0.63	0	8	25
	10	3.79	0.70	0	12	21
	Average	3.97	0.40	0	8.4	24.6
Community	11	3.76	0.66	0	12	21
	12	4.03	0.64	0	6	27
	13	4.00	0.61	1	3	29
	14	4.12	0.65	0	5	28
	15	3.94	0.86	1	10	22
	Average	3.97	0.36	0.4	7.2	25.4
Division of Labor	16	4.27	0.57	0	2	31
	17	3.76	0.66	0	12	21
	18	3.82	0.53	0	8	25
	19	3.24	0.66	4	17	12
	20	3.91	0.77	1	8	24
	Average	3.80	0.30	1	9.4	22.6
Object	21	3.88	0.70	0	10	23
	22	4.64	0.49	0	0	33
	23	4.58	0.61	0	2	31
	24	4.42	0.61	0	2	31
	25	4.45	0.67	0	3	30
	Average	4.39	0.36	0	3.4	29.6

As shown in Table 4, the average scores of each item ranged from 3.24 to 4.64, which is more than 65% of the total score, implying that students agreed to a large extent that these factors influenced their writing learning outcomes. The frequencies reveal that about 90% of participants (F = 29.6) agreed that BL contexts help them achieve goals to enhance writing performance (items 21–25). There were about three-fourths of participants agreed that the factors of subject (F = 24.6), rules (F = 24.6) and community (F = 25.4), such as motivation (item 1), interest (item 2), initiative (item 3), confidence (item 4), mood (item 5), deadline (item 6), anonymous policy (item 7), automatic evaluation criteria (item 8), grading standard (item 9), group rules (item 10), classmates and teachers (item 11–15), had positive impact on their EFL writing outcomes in the BL settings. While there were about three-fifths of participants (F = 22.6) supported that the division of labor factors, including teacher feedback (item 16), peer feedback (item 17), giving feedback (item 18), assignments (item 19) and discussions (item 20), can improve their EFL writing achievements in the BL environment.

Notably, six participants (F = 6) disagreed with the statement of item 5 (Writing on Pigai.org and discussing in WeChat groups let me feel relaxed), which is the item with the highest number of disagreements of all the statements. However, more than 60% of the participants (F≥20) agreed with items 1–4, indicating they feel motivated, interested, active, confident, and stressed in the blended writing course. In addition, item 19 (This course allows me to follow the course procedure and finish my assigned tasks easily) received the least amount of agreement (F = 12), suggesting that the assignments in the blended writing course were not easy for students to complete. Furthermore, all participants (F = 33) agreed with the statement of item 22 (This course helps me get higher grades in English writing), implying that the BL contributes to achieving the goal of getting higher writing grades.

### 4.2 Pearson correlation analysis

To further explore whether there are statistical correlations between EFL writing performance and each influencing factor and among these factors in a BL context, Pearson Correlation analysis was employed to examine the relationships between EFL writing performance and the mean scores of the influencing factors, including subject, rules, community, division of labor, and object. Table 5 illustrates the results of the Pearson Correlation analysis.

**Table 5:** Results for pearson correlation analysis of EFL writing performance and five factors.

	1	2	3	4	5	6
1.Writing performance	1					
2.Subject	0.873**	1				
3.Rules	0.608**	0.595**	1			
4.Community	0.691**	0.612**	0.448*	1		
5.Division of Labor	0.428*	0.526**	0.106	0.366*	1	
6.Object	0.625**	0.719**	0.447**	0.578**	0.350*	1

Note: ** p< 0.01. *p< 0.05.

As evident in Table 5, participants’ EFL writing performance has the strongest correlation with subject factors (r = 0.837, p<0.01), followed by community (r = 0.691, p<0.01), object (r = 0.625, p<0.01) and rules (r = 0.608, p<0.01). The most minor correlation is with division of labor (r = 0.428, p<0.05). According to Cohen’s [71] criteria for correlation coefficients, when the absolute value of R is equal to or greater than 0.5, there is a large correlation between the two variables, a medium correlation effect between 0.3 and 0.5, and a small effect size for less than 0.3. Therefore, EFL writing performance is highly correlated with subject, community, object, and rules factors while moderately correlated with division of labor.

Furthermore, the coefficient of determination (r^2^) can measure the amount of variation in the dependent variable explained by the independent variable [71]. The results indicate that the variability in students’ EFL writing achievements can be predicted by subject (r^2^ = 0.76), community (r^2^ = 0.48), object (r^2^ = 0.39), rules (r^2^ = 0.37), and division of labor (r^2^ = 0.18), with a rate of 76%, 48%, 37%, 39%, and 18%, respectively.

**Fig 2:**
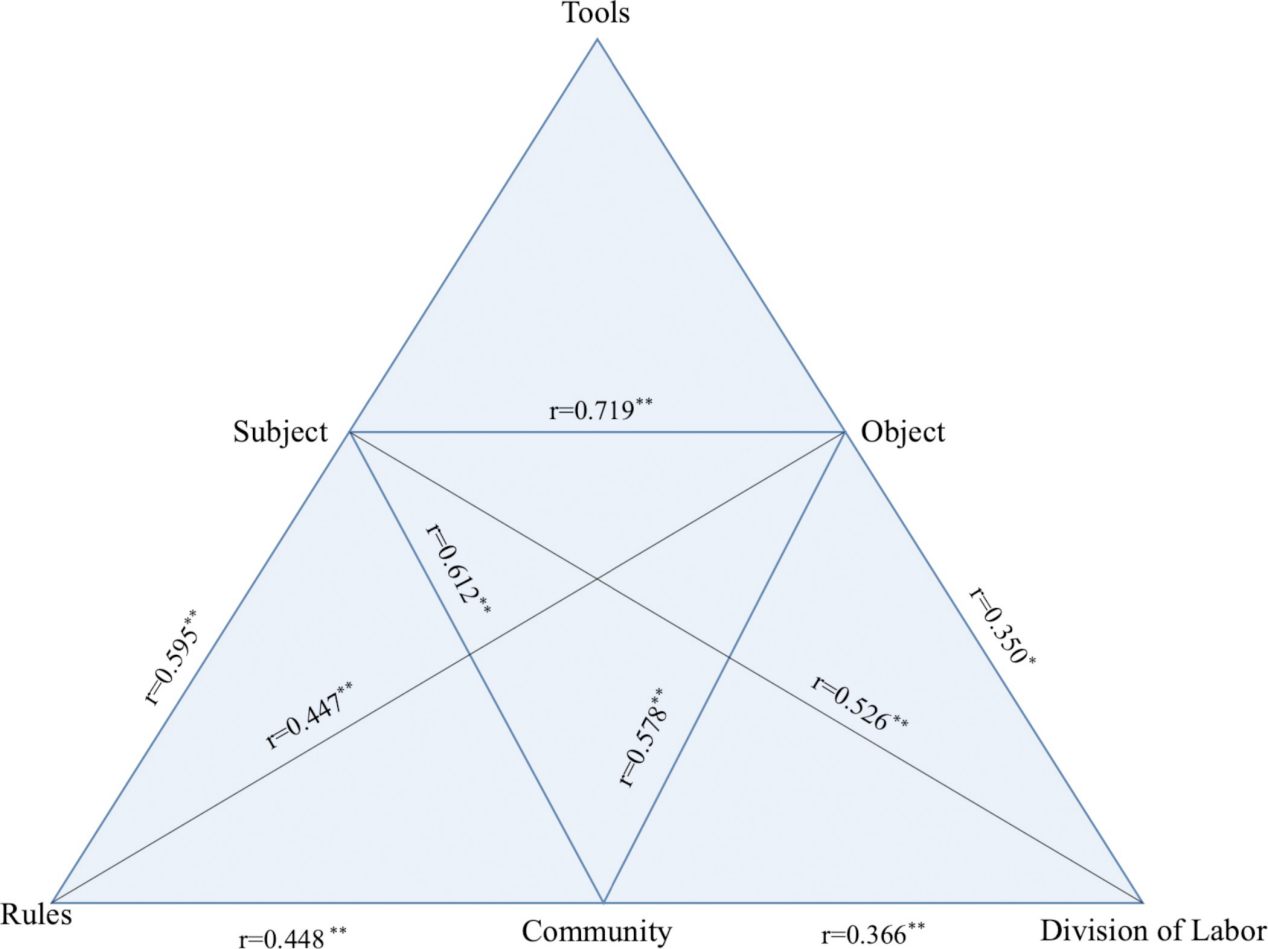
The Pearson correlation coefficients among the five influencing factors. This figure shows the Pearson correlation coefficients between the five elements, subject, object, rules, community, and division of labor, in the blended writing activity.

In addition, as presented in Table 5, the five influencing factors positively correlated with each other except for the relationship between rules and division of labor. The Pearson correlation coefficients among these factors are presented in Fig 2. Based on Cohen’s [71] criteria for correlation coefficients, subject factors, in descending order, showed enormously significant positive correlations with the factors of object (r = 0.719, p<0.01), community (r = 0.612, p<0.01), rules (r = 0.595, p<0.01), and division of labor (r = 0.526, p<0.01), respectively. Similarly, the correlation effect between community and object factors (r = 0.578, p<0.01) was considerable in the BL contexts. There were medium correlations, in descending order, between rules and community (r = 0.448, p<0.01), rules and object (r = 0.447, p<0.01), community and division of labor (r = 0.366, p<0.01), as well as object and division of labor (r = 0.350, p<0.05). However, the results indicate no direct relationship between rules and division of labor in the BL environment, as the p-value was greater than 0.05, indicating an insignificant correlation.

## 5. Discussion

The current study used AT to explore the relationship between EFL writing performance and five influencing factors and the relationship between these factors in a BL context. From the results of this study, EFL students’ argumentative writing performance is significantly and positively correlated with the influencing factors, in descending order of correlation: subject, community, object, rules, and division of labor. There is a significant positive correlation between the five factors in the BL environment, except for no direct relationship between the rules and division of labor factors. Unlike previous research [6,39,87,88] indicating that BL positively impacts EFL writing performance, the results of this study suggest that BL positively influences writing performance through different factors to different extents.

The present study found the most significant correlation between the subject factor and EFL writing performance, implying that EFL students are the most crucial element in EFL writing improvement in BL contexts. Notably, students did not feel relaxed in the blended writing course and felt the assigned tasks were not easy to complete. However, they still felt motivated, interested, active, and confident in the blended writing course as well as believed that BL can improve their EFL writing performance, suggesting that BL can provide a more challenging learning environment where students feel motivated and confident to overcome difficulties and improve their writing skills. Students may be motivated by the varied learning environments and the learning practices provided by BL as a result of feeling interested and actively engaged in their learning. This finding corroborates Anggrawan’s [89] study, which indicates that students are motivated to learn as the learning chance is provided in a different way, such as online technology.

The findings of this study, regarding the positive correlations between the subject factor and the other four factors, further explain that students’ perceived motivation, interests, active engagement and self-confidence may be affected by the expanded community (such as communicating with teachers and peers at any time and any place), effective rules (such as deadline, anonymous policy, automatic evaluation criteria, grading standard, and group rules), and beneficial division of labor (such as teacher feedback, peer feedback, giving feedback, assignments, and discussions) in the BL contexts The findings align with those of previous research in a different field such as Zheng et al. [90] in PE, Nortvig et al. [91] in Art and Craft & Design, Miranda et al. [92] in engineering education, Kemaloglu and Bayyurt [93] in pre-service teacher education, and Bhatti et al. [94] in mathematics which presented the effectiveness in breaking physical barriers and the advantage of BL in various subjects learning. Students using BL enrolled in extension activities and had additional chances to practice their skills. Also, the findings in this research reflect that of Yulianti and Sulistiyawati [95], who found that students could develop the character of discipline, responsibility, and independence through the rules in the BL environment, and the development of these characteristics could help to form a pattern of behavior in their learning which in turn help to improve academic achievement.

This research’s findings seem to differ from the results of Ma [96], who pointed out that the more peer suggestions, the lower the scores tend to be. This difference may be because peer feedback in Ma’s (ibid) study was mainly critical comments regarding content and organization of writing. Still, the peer feedback in this study contained positive and negative comments, making it easier for students to accept feedback and improve the quality of their argumentative writing. Therefore, guiding students to simultaneously praise strengths and point out weaknesses when evaluating each other’s writing is essential. However, the findings of Liu et al. [97] supported the findings in the current study that the process of completing peer assessment tasks enabled students to compare their works with others critically, and the comments from peers motivated students to revise their compositions, which led to higher writing performance. It was also found that peer feedback contributed to the quality of writing content and enhanced writing competence in Visiaty’s [98] research.

However, this study concludes that the rules in BL are not correlated with the division of labor in BL settings. This contradicts the findings of David and Victor [99], suggesting that the rules correlated with and determined the division of labor. As few studies examine the relationship between rules and division of labor in a BL context, more investigation is needed to explain this result further.

### 5.1 Implications

The findings in this study may have some related practical implications for educators and stakeholders. In general, the factors in the BL environment positively impact EFL writing performance, which provides educators with guidance for designing and implementing BL courses. Educators can focus on the influence of subject, object, rules, community, and division of labor and create personalized and flexible learning environments to enhance EFL students’ writing skills.

Specifically, according to the results of this study, subject factors have the most significant impact on writing performance in BL contexts, so teachers should conduct regular surveys to gain a deeper understanding of student’s learning needs and goals. When necessary, teachers can distribute in-class questionnaires or conduct face-to-face conversations in the classroom and then adjust their teaching strategies according to the student’s situation.

Moreover, teachers can make full use of technological means and online resources. For example, teachers can use communication applications like WeChat in EFL writing courses to provide an expanded and active community. Teachers and students can discuss and share learning materials in such applications, which encourages students to engage in learning.

Meanwhile, teachers are expected to provide specific deadlines for completing the writing, grading rules, and anonymous evaluation policies in writing courses, all of which can contribute to improving outcomes. Instructors should guide students to give critical comments and praise in peer reviews, which can make students more receptive to peer feedback and improve the quality of their writing.

Furthermore, policymakers can develop an active BL environment by providing intelligent classroom facilities, Wi-Fi coverage on campus, online course resources, and personalized learning applications. They should set up a student learning analytic system to track and analyze students’ learning and give timely feedback and advice, contributing to the quality of EFL instruction.

Finally, policymakers can provide teachers with professional training and financial assistance in implementing BL, encouraging schools and teachers to adopt BL in different subjects.

### 5.2 Limitations and recommendations

Despite the contributions outlined above, it is essential to acknowledge the limitations of this study, which should be addressed in future research. Firstly, a significant limitation of this study is that the investigation was conducted in a single university, resulting in limited sample coverage. A recommendation for future research is to survey diverse universities to achieve more comprehensive and representative data on the employment of BL in EFL writing instruction.

Secondly, the influence of different cultural, geographical, or educational backgrounds was not considered in this research, which may limit the generalizability of the study conclusions. Future research is suggested to investigate blended learning from different backgrounds to collect broader influencing factors that could promote EFL writing instruction.

Additionally, there was a limitation with the sample size. A larger sample size can improve the reliability of research results, while individual extreme values may impact the results of the study with a small sample size. Future research is recommended to apply BL among a larger sample size, and the findings might benefit from replication with a larger and more diverse sample.

Furthermore, as this study employed argumentative writing tests to assess EFL students’ writing performance, our findings may not generalize to other writing styles commonly taught in EFL courses. Future research could benefit from exploring a broader range of writing genres and provide a more comprehensive understanding of English writing instruction in a BL context.

Finally, another limitation is that the present study only collected quantitative data. Quantitative research focuses on group-level data analysis and ignores individual differences, limiting insights into individual experiences and behaviors. Further research is encouraged to incorporate qualitative methods, such as classroom observations and interviews, to provide a deeper understanding of BL’s impact on EFL students’ writing learning.

## 6. Conclusions

This research investigated the relationships between EFL writing performance and five AT-related influencing factors, as well as the relationships between these factors in a BL context, which integrates online learning and face-to-face instruction. The current study found that EFL students’ writing performance is positively and significantly correlated with factors in terms of subject, rules, community, division of labor, and object in a BL setting. Among these factors, the subject factor has the most significant impact on EFL writing performance, while the division of labor has the least in the BL context. Additionally, our findings suggest a significant positive relationship within each factor in the BL environment, except for no relationship between rules and division of labor.

The findings in the current study can offer a guide when implementing the BL approach into EFL writing curriculum plans. For instance, instructors can conduct regular surveys to understand students’ learning needs and goals better and adjust their teaching strategies accordingly. Teachers can also use applications such as WeChat to create an expanded community and encourage participation in learning. Moreover, clarifying deadlines for assignments, grading criteria, and anonymous policies on the Pigai.org platform, as well as guiding students to give critical comments and praise when conducting peer reviews, are recommended in EFL writing courses. In addition, policymakers can create a positive BL environment by providing smart classroom facilities, campus Wi-Fi coverage, and online resources. They are also suggested to set up a student learning analytic system to track students’ learning and provide timely feedback. Furthermore, policymakers can provide professional training and financial assistance to encourage educators to adopt BL in different subjects.

However, this research has limitations due to the small sample size and the reliance on quantitative data. Even though we conducted cluster sampling at a university with representative populations and carried out a rigorous instrument development process to ensure high-quality data-gathering instruments, which may enhance the accuracy of the data, increase the reliability of the findings, and mitigate these limitations, the relatively small sample size gives the impression that the results were only representing a portion of the population. Therefore, future studies may consider enlarging the sample size, involving qualitative methods, or expanding the scope of research on the impact of BL in other disciplines.

## Supporting information

S1 Checklist Inclusivity in global research. (DOCX)

S1 Appendix The questionnaire of influencing factors in blended EFL writing course. (DOCX)

S2 Appendix Writing test. (DOCX)

S3 Appendix The assessing criteria of writing performance. (DOCX)
