# Supplementary material for: Exploring the relationship between EFL students’ writing performance and activity theory related influencing factors in the blended learning context
Source: PLoS One. 2024 Jun 17;19(6):e0305668. doi: 10.1371/journal.pone.0305668 (PMC11182532; doi:10.1371/journal.pone.0305668)
Supplement: S1 Appendix — (DOCX) [file pone.0305668.s002.docx]

Appendix A

THE QUESTIONNAIRE OF INFLUENCING FACTORS IN BLENDED EFL WRITING COURSE

Dear students,

I’d like to ask you to help me by answering the following questions concerning your experience in this English Writing Course. This is not a test, so there is no “right” or “wrong” answer, and I am interested in your opinion. Please give your answers sincerely because your responses guarantee the success of the investigation. All the responses will be kept confidential. Thank you very much for your help.

**Section I: Demographic Information**

Student Number: _______ Gender:_______ Age:_________ Hometown:___________

I have learned English for _____ years.

**Section II:** **Influencing Factors**

Instructions:

Please indicate to what extent each statement is true in your case: (1) Strongly disagree; (2) Disagree; (3) Neither agree nor disagree; (4) Agree; (5) Strongly agree.

Example: If the statement is not at all true in your case, mark Strongly disagree:

|  | 1 | 2 | 3 | 4 | 5 |
| --- | --- | --- | --- | --- | --- |
| Item | Strongly disagree | Disagree | Neither agree nor disagree | Agree | Strongly agree |
| 1. I feel motivated in the blended English writing course. | √ |  |  |  |  |

|  | 1 | 2 | 3 | 4 | 5 |
| --- | --- | --- | --- | --- | --- |
| Item | Strongly disagree | Disagree | Neither agree nor disagree | Agree | Strongly agree |
| **A. Subject** | | | | | |
| 1. I feel motivated in this English writing course. |  |  |  |  |  |
| 2. Sharing my opinion in the WeChat group and writing on Pigai.org improves my learning interests. |  |  |  |  |  |
| 3. I am an active learner in this English writing course. |  |  |  |  |  |
| 4. I feel confident in this writing course. |  |  |  |  |  |
| 5. Writing on Pigai.org and discussing in WeChat groups let me feel relaxed. |  |  |  |  |  |
| **B. Rules** | | | | | |
| 6. The deadline given in Pigai.org allows me to complete the writing assignments effectively. |  |  |  |  |  |
| 7. The anonymous evaluation policy is friendly to me to promote my English writing. |  |  |  |  |  |
| 8. The automatic evaluation criteria of Pigai.org help me improve my English writing. |  |  |  |  |  |
| 9. The course grading standard helps me improve my English writing. |  |  |  |  |  |
| 10. The well-organised group helps me improve my English writing. |  |  |  |  |  |
| **C. Community** | | | | | |
| 11. I like communicating with classmates and teachers in this English writing course. |  |  |  |  |  |
| 12. This course improves communication with students and teachers, which is helpful to my learning. |  |  |  |  |  |
| 13. I can interact sufficiently with teachers in this course, which has improved my English writing skills. |  |  |  |  |  |
| 14. I can interact sufficiently with classmates in this course, which has improved my English writing skills. |  |  |  |  |  |
| 15. This course enables me to communicate with teachers and classmates at any time and space. |  |  |  |  |  |
| **D. Division of labor** | | | | | |
| 16. This course can timely deliver teacher's feedback, which help me improve my English writing skills. |  |  |  |  |  |
| 17. Peer feedback on Pigai.org helps me improve my English writing skills. |  |  |  |  |  |
| 18. Giving feedback to others on Pigai.org helps me practice my English writing skills. |  |  |  |  |  |
| 19. This course allows me to follow the course procedure and finish my assigned tasks easily. |  |  |  |  |  |
| 20. I often share ideas with classmates in this course. |  |  |  |  |  |
| **E. Object** | | | | | |
| 21. This course helps me pass examinations. |  |  |  |  |  |
| 22. This course helps me get higher grades in English writing. |  |  |  |  |  |
| 23. This course makes me invest time and effort in learning to complete targets. |  |  |  |  |  |
| 24. This course provides sufficient support to achieve my learning goals. |  |  |  |  |  |
| 25. This course has helped me master English writing skills. |  |  |  |  |  |
