# Supplementary material for: Exploring the relationship between EFL students’ writing performance and activity theory related influencing factors in the blended learning context
Source: PLoS One. 2024 Jun 17;19(6):e0305668. doi: 10.1371/journal.pone.0305668 (PMC11182532; doi:10.1371/journal.pone.0305668)
Supplement: S2 Appendix — (DOCX) [file pone.0305668.s003.docx]

Appendix B

**WRITING TEST**

Write an argumentative composition (about 250 words) on one of the following topics (45 minutes):

1. Should parents organise activities for their children’s spare time or should it be up to the children?

2. Should students spend more time participating in school activities, such as clubs and sports, or studying?

3. Do you prefer different friends or similar friends?

4. Is it better to live in your hometown after graduating from university or in a different city?
