# Supplementary material for: Exploring the relationship between EFL students’ writing performance and activity theory related influencing factors in the blended learning context
Source: PLoS One. 2024 Jun 17;19(6):e0305668. doi: 10.1371/journal.pone.0305668 (PMC11182532; doi:10.1371/journal.pone.0305668)
Supplement: S3 Appendix — (DOCX) [file pone.0305668.s004.docx]

Appendix C

THE ASSESSING CRITERIA OF WRITING PERFORMANCE

| Numeric Grade | Grade Descriptor | Writing Assessment Points | | | | |
| --- | --- | --- | --- | --- | --- | --- |
|  |  | Content (30) | Style (20) | Language use (25) | Organisation (20) | Mechanics (5) |
| 85-100 | Outstanding | ﻿Substantial, specific, and/ or illustrative content demonstrating strong development and sophisticated ideas  (26-30) | ﻿Precise, illustrative use of various words and sentence structures to create a consistent writer’s voice and tone appropriate to the audience.  (18-20) | ﻿Native-like fluency in English grammar; correct use of relative clauses, prepositions, modals, articles, verb forms, and tense sequencing; no fragments or run-on sentences  (17-25) | ﻿Sophisticated arrangement of content with evident and/or subtle transitions.  (19-20) | The use of English writing conventions is correct and very neat.  (5) |
| 75-84 | Excellent | ﻿Sufficiently developed content with adequate elaboration or explanation.  (23-26) | Generic use of a variety of words and sentence structures that may or may not create the writer’s voice and tone appropriate to the audience.  (16-18) | ﻿Advanced proficiency in English grammar; some grammar problems don’t influence communication, although the reader is aware of them; no fragments or run-on sentences.  (15-17) | ﻿Functional arrangement of content that sustains a logical order with some evidence of transitions.  (17-19) | Some problems with writing conventions, the paper is neat and legible.  (4) |
| 60-74 | Good | ﻿Limited content and inadequate elaboration of explanation.  (18-23) | Limited word choice and control of sentence structures that inhibit voice and tone.  (14-16) | ﻿Ideas are getting through to the reader, but grammar problems are apparent and have a negative effect on communication, run-on sentences or fragments present  (11-15) | ﻿Confused or inconsistent with arrangement of content with or without attempts at transition.  (14-17) | Uses general writing conventions but has errors; acceptable to read.  (3) |
| 0-59 | Fail | ﻿Superficial and/or minimal content.  (0-18) | Minimal variety in word choice and minimal control of sentence structure.  (0-14) | ﻿Numerous serious grammar problems interfere with the communication of the writer’s ideas; a grammar review of some areas clearly needed; difficult-to-read sentences.  (0-11) | ﻿Minimal control and content arrangement.  (0-14) | Completed disregard for English writing conventions, paper illegible.  (0-2) |
|  | Insufficient to score, off-topic, blank paper, or written predominantly in another language. | | | | | |
